# Molecular analysis of T-cell Acute Lymphoblastic Leukemia arising after Essential Thrombocythemia foreshadows a distinct clonal route for lymphoid blast crisis in Philadelphia-negative chronic myeloproliferative neoplasm: a case report with literary review

**DOI:** 10.1007/s00277-025-06404-z

**Published:** 2025-05-22

**Authors:** Francesco Grimaldi, Mara Memoli, Simona Avilia, Roberta Russo, Giulia Scalia, Roberta Visconti, Santa Errichiello, Barbara Izzo, Fabrizio Pane

**Affiliations:** 1 Department of Clinical Medicine and Surgery, Hematology Division, University of Napoli “Federico II”, Naples, Italy; 2 CEINGE Biotecnologie Avanzate, Naples, 80145 Italy; 3 Department of Molecular Medicine and Medical Biotechnology, University of Naples Federico II, Naples, Italy

**Keywords:** Secondary Acute Lymphoblastic Leukemia, Blast crisis, Clonal hematopoiesis

## Abstract

Progression to Acute Myeloid Leukemia is a well-known complication of classical philadelphia-negative chronic myeloproliferative neoplasms, while disease progression to Acute Lymphoblastic Leukemia remains an extremely unfrequent event. A molecular explanation for this rare phenomenon is missing. However, the clonal haematopoiesis mostly present in these patients may work as a seeding soil for a second neoplastic disease. Molecular results from this case study reporting a secondary Acute Lymphoblastic Leukemia presenting after Essential Thrombocythemia support this hypothesis. In this contest secondary Acute Lymphoblastic Leukemia should not be considered as real blast crisis, but rather as a second cancer determined by a different clonal route. Given the unique features of this case, a review of the published cases of MPN transforming to Acute Lymphoblastic Leukemia available in literature is provided in the article.

**Supplementary Information:**

The online version contains supplementary material available at 10.1007/s00277-025-06404-z.

## Introduction

Progression to Acute Myeloid Leukemia (AML) is a well-known complication of classic Philadelphia-negative chronic myeloproliferative neoplasms (MPN) including Polycythaemia Vera (PV), Essential Thrombocythemia (ET) and Primary Myelofibrosis (PMF) [1]. The incidence of leukemic transformation varies across MPN subtypes, reaching 10–20% at 10 years in PMF, followed by PV, where incidence is reported about 2.3% at 10 years and 7.9% at 20 years, and finally in ET, where transformation risk is expected to be less than 3% at 10 years, predominantly in cases later reclassified as prefibrotic PMF [2–4]. Currently, *JAK2*, *CALR* and *MPL* mutations are considered to play a pivotal role as driver events in MPN pathogenesis [5], while leukemic evolution is commonly associated with accumulation of additional mutations in different myeloid cancer genes, such as *RUNX1*, *ASXL1*, *IDH2*, and *TP53*, that contribute to increase clonal complexity and heterogeneity in the hematopoietic stem cell (HSC) compartment [6, 7].

In contrast, transformation to secondary Acute Lymphoblastic Leukemia (sALL) is unusual and exceedingly rare in MPNs [8]. Due to paucity of molecular and clinical data available [9, 10], mechanism of lineage switch from a chronic MPN to a lymphoid blast crisis remains elusive. Given that most of reported cases have been associated with B-lineage ALL and are associated with *JAK2* positivity [9, 10], and that *JAK2* mutation has been detected in B/NK/T-lymphoid precursors from PV and PMF patients [11, 12], it has been speculated that the aberrant *JAK2* tyrosine kinase activity in a common myelo-lymphoid CD34 + progenitor may play a causative role and contribute to lymphoid transformation. Nevertheless, some studies [13, 14] have described sALL cases harboring a molecular profile completely distinct from the antecedent MPN, implying independent clonal origins.

In according with these observations, we report the case of a patient with *JAK2-V617F*–positive ET who developed more than a decade later a T-cell sALL. Molecular analyses performed during the disease course clearly revealed a *JAK2*-negative lymphoid clone arising from a persistently *DNMT3A*-mutated clonal background, supporting a model in which clonal hematopoiesis (CH) may contribute to a dual neoplastic evolution, with a different clonal route for the rare sALL diagnosed after MPNs.

## Case description

In October 2012 a 62 year-old women was initially referred to our clinic for thrombocytosis. *JAK2-V617F* molecular test (Supplementary methods, S1) was positive on peripheral blood and a bone marrow biopsy confirmed the histological diagnosis of ET.

**Fig. 1:**
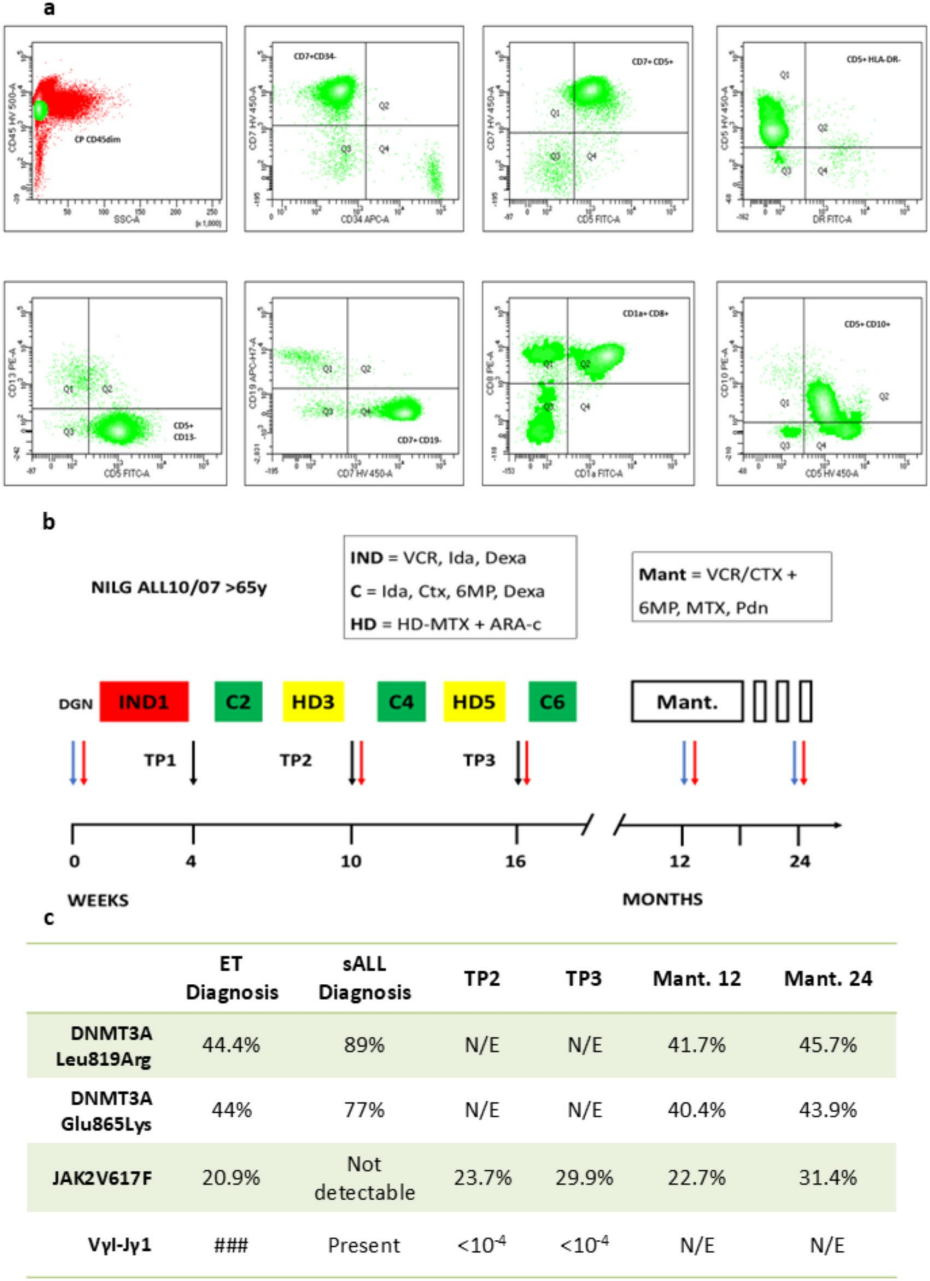
**a**: Diagnosis of secondary T-ALL (sALL). **b**: NILG-ALL 10/07 regimen. IND = induction, C = consolidation, HD = high dose chemotherapy, Mant = maintenance. VCR = vincristine, IDA = idarubicin, Dexa = Dexamethasone, Ctx = cyclophosphamide, 6MP = 6-mercaptopurin, MTX = Methotrexathe, PDN = prednisone. Arrows = bone marrow sampling; Black arrow = MRD sampling, Red arrow = JAK2 allelic burden, Blue arrow = NGS sampling. **c**: mutations kinetics observed from ET diagnosis trough sALL treatment

Patient was started on hydroxyurea, and continued on with good response and without thrombotic events over the following ten years. A decade later, she was urgently admitted with high fever, fatigue and joint pain. Complete blood counts at time showed: Hgb 10,4 g/dl, WBC 20.680/mm3, PLT 23.000/mm3. Peripheral blood and bone marrow smear revealed lymphoid blast cells (80% and 95% respectively), with following surface markers at flow-cytometry analysis: CD45dim, DR-, CD34-, CD5+, CD7+, CD2+, CD8+, CD1a+, CD99+, CD10+, CD3-, cyCD3+/- (Supplementary methods, S3). According to EGIL classification, diagnosis of secondary Cortical T-ALL was made (Fig. 1a). Standard cytogenetic, with FISH study to detect chromosome 9, 11 and 17 aberrations, revealed a normal karyotype (46 XX), while molecular testing by RT-PCR for *SIL/TAL*, *NUP98* rearrangments and *TAF1/NUP214* was negative. Remarkably, the *JAK2-V617F* mutation became undetectable in the bone marrow at diagnosis of sALL. With RT-PCR, a disease specific VγI-Jγ1.1 *IGH* sequence was identified for monitoring of minimal residual disease (MRD) (Supplementary methods, S1). Patient started induction chemotherapy according to NILG 10/07 ALL protocol [15] for elderly patients (Age > 65 years). Bone marrow evaluation performed after cycle 1, 3 and 5 of chemotherapy (Time point 1, 2, 3; see Fig. 1b) showed complete remission (CR), with molecular MRD constantly negative (below < 1 × 10-4 level of test sensitivity). Interestingly, JAK2-V617F mutation became again detectable in the bone marrow, with an increase in allele burden of 23,7% at Tp2 and 29,9% at Tp3. Patient successfully completed the planned 6 cycles of chemotherapy and started maintenance therapy. Bone marrow evaluation performed after first and second year of maintenance confirmed the CR status, with no blast cells detected by flow-cytometry, and *JAK2-V617F* positivity with a stable allele burden of 22,7% and 31.4% respectively. To further investigate the clonal origin of the T-cell ALL, a Next-Generation Sequencing (NGS) analysis using a 36-gene myeloid panel (Supplementary methods, S2) was performed on bone marrow samples collected and previously stored at four time points: ET diagnosis, sALL diagnosis, and during first- and second-year maintenance. NGS analysis revealed two missense mutations in *DNMT3A* gene, occurring on exon 21 (Leu819Arg; T2456 > G) and exon 22 (Glu865Lys; G2593 > A), known to be pathogenic and related with clonal haematopoiesis (CH). Interestingly, these two mutations were already detectable at the time of ET diagnosis, with a variant allele frequency (VAF) of 44.4% (for exon 21 mutation) and 44% (for exon 22 mutation). Their VAFs increased to 89% and 77% at the time of T-ALL diagnosis, respectively. Despite intensive chemotherapy, the VAFs returned to relatively stable levels of 41.7% (exon 21) and 40.4% (exon 22) after the first year of maintenance, and of 45.7% (exon 21) and 43.9% (exon 22) after the second year (see Fig. 1c). Finally, to assess penetrance of *DNMT3A* and *JAK2* mutations in pre-leukemic HSC compartment, NGS analysis was performed on sorted B and T-lymphocytes in peripheral blood, even if only from peripheral blood collected at second year of maintenance (Supplementary methods, S2, S3). Very interestingly, no *JAK2* mutations were detected in either lineage. However, both *DNMT3A* mutations were identified in T-cells, even if with a low allele frequency below limits of assay detection (VAF below 5%), suggesting its persistence in early hematopoietic progenitors (Supplementary Methods, S2–S3).

## Discussion with literature review

Several studies have reported the incidence of secondary acute lymphoblastic leukemia (sALL) following a prior malignancy. A large SEER analysis [16] found that 6.6% of newly diagnosed ALL cases in the United States occurred as secondary malignancies. A retrospective series from the GIMEMA group reported a lower incidence of 2.3% [17], while Aldoss et al. [18] more recently described an incidence of 9.3% at a single U.S. institution. In all these cohorts, fewer than 10% of sALL cases followed a primary hematologic malignancy, and transformation from MPN to sALL remains a rare and anecdotal event. Given the rarity of this case, we conducted a literature review of published cases of MPN transforming to ALL. We searched PubMed using the following keywords: “acute lymphoblastic leukemia, myeloproliferative neoplasm, chronic myeloproliferative, essential thrombocythemia, polycythemia vera, myelofibrosis”. After removing reviews and other irrelevant papers, we identified a total of 31 patients reported in literature from 1978 to the present, including the current case. The complete patient series is reported in Table 1 with references.

Across these 31 identified patients, median time to leukemic transformation from initial diagnosis of MPN was of 10 years. In 15 patients a molecular analysis of MPN phase was available, with a *JAK2V617F* mutation identified in 66% of cases (10/15). The major part of patients (78%) showed a B-cell phenotype after leukemic transformation (24/31), and, despite intensive chemotherapy, only 25% of patients resulted alive at reported follow-up. Prevalence of sALL incidence in *JAK2*-mutated MPN have stimulated the idea that a common *JAK2*-mutated myelo-lymphoid precursor could explain the origin of this condition, sustained by the experimental evidence that generally a low *JAK2* allelic burden (3–5%) can be found both in B- and T-lymphocytes of MPN patients [10, 11].

However, in our case *JAK2-V617F* canonical mutation disappeared at the sALL onset, and became again detectable after the patient obtained the CR, with an allele burden progressively increased during maintenance therapy. The disappearance of JAK2V617F at in the moment of diagnosis, when the blast size in bone marrow is relevant, have been already described even with differently mutated MPN (*CALR*-mut or *MPL*-mut; see Table 1) and strongly suggests that the leukemic transformation may occur independently of the original MPN-defining mutation. These findings finally align with previous molecular case studies indicating distinct clonal origins for MPN and sALL phases [13, 14].

**Table 1:** Summary of cases of MPN transformed into sALL reported in literature

Case no.	Year	Age / gender	MPN subtype	Molecular status	MPN treatment	Time to progression (years)	s-ALL Phenotype	Cytogenetics (of sALL)	Clinical outcome	Reference
1	1978	77 / M	PV	NA	Phlebotomy, CHL	6	B-cell	Normal	Dead	[19]
2	1980	61 / M	PMF	NA	Oxymetholone, splenectomy	5	B-cell	Aneuploid	Dead	[20]
3	1980	53 / F	PPV-MF	NA	Phlebotomy	18	Burkitt’s	NA	Dead	[20]
4	1986	74 / M	PV	NA	P32, Bu, CHL, BCNU	6	Null	NA	Dead	[21]
5	1987	42 / M	PV	NA	P32, Bu	10	Null	Del 6q, +8	Dead	[22]
6	1987	20 / M	PV	NA	P32, Bu	10	T-cell	NR	Dead	[22]
7	1988	68 / F	PV	NA	P32, Bu, CHL	25	B-cell	Complex	Dead	[13]
8	1993	54 / F	PV	NA	P32	11	B-cell	t(9;22)	Dead	[23]
9	1994	76 / M	PV	NA	BU	16	B-cell	Diploid	Dead	[24]
10	1995	50 / M	ET	NA	HU	3	B-cell	Complex	Alive	[25]
11	1996	54 / F	PV	NA	P32, HU	13	B-cell	NA	Alive	[26]
12	1996	87 / F	ET	NA	HU	1	T-cell	NA	NA	[27]
13	1996	70 / F	ET	NA	P32, HU	19	B-cell	Diploid	NA	[28]
14	1999	63 / M	PPV-MF	NA	P32, HU	6	B-cell	NA	Dead	[29]
15	2005	53 / M	PMF	NA	HU	2	B-cell	NA	Dead	[30]
16	2012	56 / M	PMF	Neg	HU	1	B-cell	t(9;22), del 20q	Dead	[31]
17	2012	54 / M	PPV-MF	Exon 12*	Phlebotomy	4	B-cell	Diploid	Dead	[32]
18	2014	5 / F	PV	NA	Phlebotomy	17	B-cell	NA	NA	[33]
19	2014	67 / F	ET	V617F*	HU	16	B-cell	t(9;22)	Dead	[14]
20	2015	65/ M	ET	V617F	NA	16	B-cell	Del (9) (p13)	NA	[34]
21	2015	59 / M	ET-MF	V617F	NA	10	B-cell	Del 13q and 20q	NA	[34]
22	2015	72 / M	PV	V617F	HU	3	B-cell	Normal	Alive	[35]
23	2016	64 / F	PV	V617F	HU	4	B-cell	Normal	Alive	[36]
24	2016	65 / F	ET	CALR	HU	3.5	B-cell	Hyperdiploid	Alive	[37]
25	2017	69 / F	ET	CALR	HU, anagrelide, ruxolitinib	26	B-cell	t(1;19)	Dead	[38]
26	2017	66 / F	PMF	V617F	HU, lena	11	B-cell	Complex monosomal	Dead	[39]
27	2018	76 / M	PPV-MF	V617F*	Phlebotomy, ruxolitinib	7	B-cell	NA	NA	[40]
28	2020	62 / M	PMF	V617F	Epo	3	T-cell	i(17)	Dead	[41]
29	2021	49 / M	ET	MPL	HU	10	T-cell	Complex	Alive	[42]
30	2021	67 / F	ET	MPL*	HU	13	B-cell	t(9;22)	NA	[43]
31	2022	77 / F	ET	Triple-neg	HU	2	B-cell	NA	Alive	[44]
32	2024	62 / F	ET	V617F*	HU	10	T-cell	Normal	Alive	Current Case

ET, essential thrombocythemia; PV, policythemia vera; PMF, primary myelofibrosis; PPV-MF, post-PV myelofibrosis; PET-MF, post-ET myelofibrosis; NA, not available in the report; HU, hydroxyurea; CHL, clorambucil; P32, Phosphorus-32; Bu, busulphan; *Mutation not detectable, or with VAF ≤ 5%, at leukemic transformation

*JAK2*-driven hemopoiesis re-expanded once the patient achieved CR, showing the ability of *JAK2*-mutated HSC to survive under stress conditions as ones given by chemotherapy, and as highlighted in several MPN disease models [45, 46]. NGS analysis identified two *DNMT3A* gene mutations that were present at the time of diagnosis of ET, increased in VAF only at the diagnosis of T-cell sALL, and then remained relatively stable during the entire course of the patient monitoring. Several studies of tumoral DNA from T-lymphoproliferative neoplasm [47, 48], and recently even from elderly patients with T-cell ALL [49, 50], have clearly identified an increased incidence of myeloid gene mutations related to CH, such as *DNMT3A* and *TET2*, in leukemic cells. In particular, Bond et al. [50], have analyzed implications of *DNMT3A* mutations in T-cell ALL, performing a comprehensive genetic and clinico-biological analyses of T-ALL treated during the GRAALL-2003 and − 2005 studies. They were able to show that, at least in two patients, *DNMT3A* mutations were present in non-leukemic cell DNA, providing the first potential evidence of age-related CH in T-cell acute lymphoblastic leukemia, and that *DNMT3A* mutation was associated with older age. In addition to this, very recently preliminary results from Horns et al. [51] showed that CH mutations are detectable in secondary B-cell ALL, and define a particular group of sALL diagnosed in patients exposed to lenalidomide for Multiple Myeloma treatment. Also the mutational profile of these patients showed a strong enrichment of CH genes in ALL, with three mutually exclusive patterns: *TP53* mutated, *IDH2* mutated and *DNMT3A* (Including non-R882) mutated. Altogether these data suggest that *DNMT3A* driven CH may contribute to ALL development, at least in older adults or in secondary ALL.

In according with these finding, in our patient the *DNMT3A* mutations were present at higher VAF (40%) than the *JAK2-V617F* mutation (20,9%) at the time of ET diagnosis, suggesting that the *DNMT3A* mutation occurred as first in the HSC compartment. This condition is concordant with experimental observations that determined the mutational hierarchy in which the HSC can acquire canonical *JAK2* mutation [52]. In some cases, mutations in epigenetic regulators such as *TET2* can be acquired before *JAK2*, and in these conditions the HSC compartment is completely dominated by *TET2* single-mutant cells (*TET2*-first). NGS data from our patient are consistent with the *TET2*-first model of MPN (even if in our case the mutations involved a different epigenetic regulator as *DNMT3A*) and suggest that the HSC compartment was dominated by *DNMT3A* at the time of diagnosis of ET. Two other elements support this idea. Firstly, *DNMT3A* mutations has been detected at a very low level even in mature T-lymphocytes in our sorting experiment, suggesting its persistence in a hierarchically higher progenitor of HSC. Secondly, the *DNMT3A* mutation remained detectable in bone marrow during CR, similarly to what happens in AML patients, confirming their nature of CH mutations [53, 54].

**Fig. 2:**
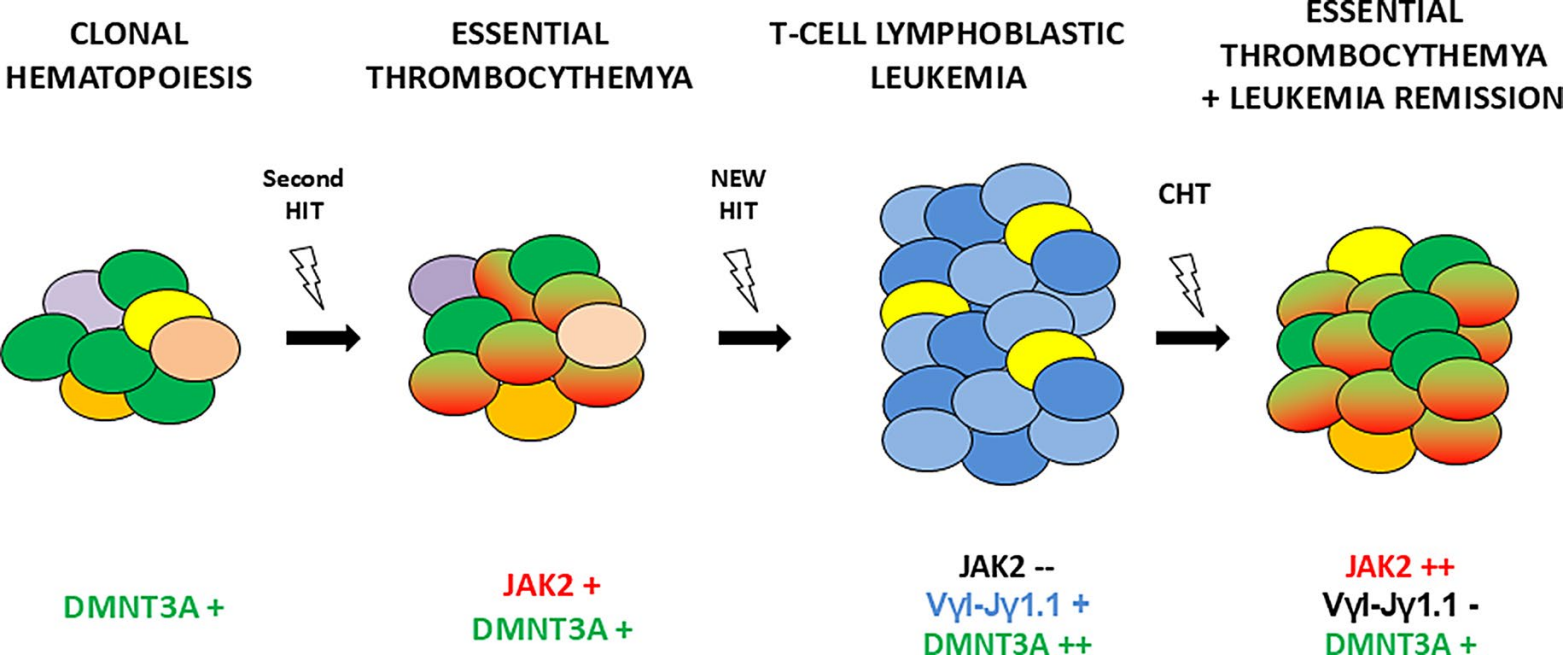
ET originates from clonal haematopoiesis when JAK2V617F mutation occurred on DNMT3a mutated cells. Years after, a new genetic hit selects a s-ALL clone and drives leukemia expansion over JAK2-mutated cells. After chemotherapy, under stress conditions, DNMT3a + JAK2 + clone re-expanded over polyclonal haematopoiesis, with an increasing allele burden

Collectively, these data suggest that in our patient, an early *DNMT3A*-driven CH event created a clonal foundation that first facilitated the development of ET via *JAK2* acquisition, and later, after an uncharacterized second genetic hit, led to the emergence of T-ALL (Fig. 2). However, once chemotherapy cleared all the blast cells in the bone marrow, the *DNMT3A/JAK2* clone re-emerged and expanded under hematopoietic stress conditions. These findings support a model in which lymphoid blast crisis in MPNs arises from a distinct clonal trajectory independent of *JAK2*, *CALR*, or *MPL* mutations, yet rooted in a common CH background, as has been noted in other conditions [55, 56].

## Conclusions

Philadelphia chromosome-negative MPNs are characterized by the presence of driver mutations (*JAK2*, *CALR*, and *MPL*), which play a central role in disease classification and pathogenesis. However, the importance of screening for additional somatic mutations in genes involved in epigenetic regulation (i.e. *ASXL1*, *TET2*, *DNMT3A*), RNA splicing (*SRSF2*, *U2AF1*, *SF3B1*), and signaling pathways (*NRAS*, *KRAS*, *SH2B3*) has become more relevant, considering their association with disease progression, leukemic transformation, and overall survival. Incorporating comprehensive molecular profiling into routine clinical evaluation enhanced in recent years diagnostic accuracy, risk stratification, and personalized treatment strategies with refined prognostic models such as MIPSS70 + and GIPSS [57]. Several of these mutations also occur in the context of CH, condition characterized by age-related acquisition of somatic mutations in HSC in the absence of overt hematologic malignancy. The overlap between CH-associated mutations and those found in MPNs suggests a continuum of clonal evolution, with early mutations potentially influencing MPN development and subsequent disease trajectory. In this view, the clonal dynamics tracked down from our study shed some lights on the mechanism of lymphoid blast crisis diagnosed in the contest of Philadelphia chromosome-negative MPNs. The disappearance of *JAK2* mutation during sALL, followed by its re-emergence post-therapy, and the persistence of *DMT3A* mutation, strongly support the hypothesis of divergent clonal evolution from a common CH background. In this contest sALL observed after Philadelphia-negative MPN should not be considered as a real blast crisis, but rather as a second neoplasm originating from a shared clonal origin. Despite the rarity of these cases, additional genomic studies, possibly at a single-cell-level, will be needed to clearly understand and finally assess the origin of this rare phaenomenon, refining our understanding of clonal architecture in MPNs evolution.

## Electronic supplementary material

Below is the link to the electronic supplementary material.

Supplementary Material 1

## Data Availability

No datasets were generated or analysed during the current study.
