# Supplementary material for: Molecular analysis of T-cell Acute Lymphoblastic Leukemia arising after Essential Thrombocythemia foreshadows a distinct clonal route for lymphoid blast crisis in Philadelphia-negative chronic myeloproliferative neoplasm: a case report with literary review
Source: Ann Hematol. 2025 May 22;104(5):2897–904. doi: 10.1007/s00277-025-06404-z (PMC12141130; doi:10.1007/s00277-025-06404-z)
Supplement: Supplementary file 1 — Supplementary Material 1 [file 277_2025_6404_MOESM1_ESM.docx]

**Supplementary methods**

**Supplemental 1 (S1). Real-time PCR assay**

The JAK2V617F mutational load was analyzed by Allele-specific real-time PCR, using JAK2 MutaQuant assay (Ipsogen) that detects the JAK2 V617F allele in genomic DNA by real-time detection of fluorescent signals using double-dye hydrolysis oligonucleotide probes. Calibration standards at 4 different concentrations were included with each PCR run, and wild-type and mutated alleles were detected in 2 separate reactions for the same sample. Use of a standard curve in result analysis allowed precise quantitation of mutant and wild-type alleles. The results were expressed as the percentage of mutant allele in the total reaction mixture. Each sample was tested in duplicate using ABI PRISM 7900HT SDS detection system (Applied Biosystem). The results were expressed as the percentage of mutant allele CN in the total CN [(CNV617F/CNV617F+CNWT)×100]. The background level or level of blank (LOB) was determined on negative samples, this was found to be 0.014%. The limit of detection (LOD) was determined using samples that were known to be positive but with low expression and this was found to be 0.061%, with a 90% confidence interval upper bound at 0.091%. This optimal sensitivity can be obtained on specimens containing at least 10,000 copies of the JAK2 gene (wild-type or V617F mutation).

Minimal residual disease was carried out by real-time quantitative-PCR (RQ-PCR) for T-cell receptor (TR) genes rearrangements. RQ-PCR targets were tested for speciﬁcity and sensitivity with the aim of selecting for each patient two markers with a sensitivity of at least 10-4 and a quantitative range of at least 10-4, with a molecular probe recognizing patient-specific T-receptor gene rearrangement. Analysis was processed at EuroMRD-certified GIMEMA laboratory in Rome (Università La Sapienza) trough MRD-ITALLab platform, and performed according to the EuroMRD guidelines.

**Supplemental 2 (S2). Next-generation sequencing analysis**

Next-generation sequencing (NGS) analysis was performed using SOPHIA Genetics Myeloid Solution panel (Sophia Genetics, Saint Sulpice, Switzerland), covering 30 genes (ABL1, ASXL1, BRAF, CALR, CBL, CEBPA, CSF3R, DNMT3A, ETV6, EZH2, FLT3, HRAS, IDH1, IDH2, JAK2, KIT, KRAS, MPL, NPM1, NRAS, PTPN11, RUNX1, SETBP1, SF3B1, SRSF2, TET2, TP53, U2AF1, WT1, ZRSR2). Samples were first diluted with IDTE (10 mM Tris, 0.1 mM EDTA) buffer to obtain 200 ng of DNA in 30 μL of final volume, and then library preparation was initiated with enzymatic DNA fragmentation, followed by barcode adapter ligation at the ends of fragments and purification by AMPure beads (Beckman Coulter, Milan, Italy) to remove enzymatic reaction components and unbound adapters. Subsequently, AMPure beads were used for dual size selection of fragments of length between 300 and 700 bp, and the library was then amplified by PCR. After a further purification step, libraries were quantified and subjected to quality control using a 4200 Tape Station System (Agilent, Milan, Italy). Samples were further processed only if concentrations were 5–20 ng/μL and fragment length of 300–700 bp. Next, 150 ng of each library were pooled, 2 μL of universal blockers were added to prevent non-specific hybridization between adapter sequences, and 5 μL of human cot DNA for binding repetitive sequences were mixed to avoid secondary DNA structure formation. Lyophilized DNA samples were obtained using a vacuum pump (Savant DNA SpeedVac, Thermo Scientific, Milan, Italy), and then resuspended in an appropriate volume using the hybridization mix. Subsequently, DNA denaturation was carried out at 95°C for 10 min, then target-specific biotinylated probes were added, and hybridization was performed at 65°C for 4 h. After this step, streptavidin-coated beads were used for DNA capture, unbound fragments were removed by washing, samples were subjected to amplification by PCR, and then to a second purification step. Afterwards, DNA quality and concentration were monitored using a 4200 TapeStation System (Agilent), and libraries were diluted to obtain a final concentration of 4 nM. Next, further sample dilution and denaturation were performed according to manufacturers' instructions, and 10 pM of denatured library and 5% of PhiX Control v3 Library (Illumina, Milan, Italy) were loaded in the flow cell for sequencing.

**Supplemental 3 (S3). Flow-cytometry analysis and B- and T-lymphocyte sorting**

For immunophenotyping, 50 μL of fresh heparinized whole PB or BM was stained at 4°C in dark for 30 minutes in the presence of appropriate amounts of monoclonal antibodies as previously described. For characterization of PB or BM mononucleated leukemic cells, the following antibodies were employed CD2, CD5, CD3, CD7, CD4, CD8, CD1a, CD99 CD19, CD10, CD24, CD20, CD22, CD13, CD33, CD117, CD11a, CD11b, CD11c, CD36, CD14, CD16, CD56, CD66b, CD64, CD61, CD42b, CD34, HLA-DR, CD38, CD71, CD25, CD69, CD45RA, CD45RO, CD15, cCD79a, MPO-7, TdT, cCD3 (from Beckman Coulter, Marseille Cedex 9, France and from BD San Diego, CA, USA). Blast cells were gated by using CD45 vs SSC-A dot-plot. The Blasts showed the following phenotype: CD45dim, DR-, CD34-, CD5+, CD7+, CD2+, CD4+, CD8+, CD1a+, CD99+, CD10+, CD3-,cyCD3+/-. Red cell lysis was performed with ammonium chloride lysing solution (Pharmlyse; BD Bioscience, San JOSE, CA), at room temperature for 10 minutes washed twice with phosphate-buffered saline (PBS) and finally resuspended in 500 μL PBS for acquisition. At least 200 000 events were recorded.Samples were acquired on Becton Dickinson Facs Canto II cytometer (BD Bioscience, San JOSE, CA) and post-acquisition analysis was carried out using BD Facs Diva software. The lower level of detection was 10^-4^ (as such, zero corresponds to a level below 1/10000 cells). The values have been expressed as a percentage. External quality control was performed using UK NEQAS for Leucocyte Immunophenotyping tests (<https://ukneqas.org.uk/>).

For the sorting experiment, mononucleated cells (MNCs) were isolated after lyses. Peripheral blood lymphocytes were labeled by incubation using antibodies CD3 specific for T lymphocytes and CD19 for B lymphocytes, with a purity check ≥98% performed by FACS re-analysis. DNA from sorted lymphocytes was obtained using the QIAmp DNA blood kit (Qiagen GmbH) and processed trough Next-generation sequencing (NGS) analysis with SOPHIA Genetic platform as already described.
